# Left ventricular remodelling in rheumatic heart disease – trends over time and implications for follow-up in childhood

**DOI:** 10.1186/s12872-023-03497-0

**Published:** 2023-09-15

**Authors:** Bradley MacDonald, Adrian Tarca, Louise Causer, Katie Maslin, Di Bruce, Rachel Schreiber-Wood, Mohit Kumar, James Ramsay, David Andrews, Charley Budgeon, Judith Katzenellenbogen, Asha C. Bowen, Jonathan Carapetis, Mark K. Friedberg, Deane Yim

**Affiliations:** 1 Children’s Cardiac Centre, Department of Infectious Diseases, Perth Children’s Hospital, 15 Hospital Ave, Nedlands, Perth, WA 6008 Australia; 2 Labatt Family Heart Center, Hospital for Sick Children and University of Toronto, Toronto, ON Canada; 3 School of Population and Global Health, University of Western Australia, Perth, Australia; 4 Wesfarmers Centre for Vaccines and Infectious Diseases, Telethon Kid’s Institute, University of Western Australia, Perth, Western Australia

**Keywords:** Rheumatic heart disease (RHD), Left ventricular remodelling, Left ventricle volume

## Abstract

**Background:**

Rheumatic heart disease (RHD) is the most common form of acquired heart disease worldwide. In RHD, volume loading from mitral regurgitation leads to left ventricular (LV) dilatation, increased wall stress, and ultimately LV dysfunction. Improved understanding of LV dynamics may contribute to refined timing of intervention. We aimed to characterize and compare left ventricular remodelling between rheumatic heart disease (RHD) severity groups by way of serial echocardiographic assessment of volumes and function in children.

**Methods:**

Children with RHD referred to Perth Children’s Hospital (formally Princess Margaret Hospital) (1987–2020) were reviewed. Patients with longitudinal pre-operative echocardiograms at diagnosis, approximately 12 months and at most recent follow-up, were included and stratified into RHD severity groups. Left ventricular (LV) echocardiographic parameters were assessed. Adjusted linear mixed effect models were used to compare interval changes.

**Results:**

146 patients (median age 10 years, IQR 6–14 years) with available longitudinal echocardiograms were analysed. Eighty-five (58.2%) patients had mild, 33 (22.6%) moderate and 28 (19.2%) severe RHD at diagnosis. Mean duration of follow-up was 4.6 years from the initial diagnosis. Severe RHD patients had significantly increased end-systolic volumes (ESV) and end-diastolic volumes (EDV) compared to mild/moderate groups at diagnosis (severe versus mild EDV mean difference 27.05 ml/m^2^, p < 0.001, severe versus moderate EDV mean difference 14.95 ml/m^2^, p = 0.006). Mild and moderate groups experienced no significant progression of changes in volume measures. In severe RHD, LV dilatation worsened over time. All groups had preserved cardiac function.

**Conclusions:**

In mild and moderate RHD, the lack of progression of valvular regurgitation and ventricular dimensions suggest a stable longer-term course. Significant LV remodelling occurred at baseline in severe RHD with progression of LV dilatation over time. LV function was preserved across all groups. Our findings may guide clinicians in deciding the frequency and timing of follow-up and may be of clinical utility during further reiterations of the Australia and New Zealand RHD Guidelines.

**Supplementary Information:**

The online version contains supplementary material available at 10.1186/s12872-023-03497-0.

## Background

Rheumatic heart disease (RHD) following acute rheumatic fever (ARF) remains the most common form of acquired heart disease worldwide and has significant associated morbidity and mortality [1, 2]. RHD is characterized by pathological valvular regurgitation on echocardiogram, in an individual with sufficient clinical symptoms and signs for an ARF diagnosis [3, 4]. Patients with RHD are followed serially with echocardiograms to monitor the severity of valvular involvement and to appropriately time surgical intervention if required [5]^5^. Risk stratification for RHD currently considers valvular regurgitation and stenosis but does not consider left ventricular (LV) changes secondary to valvular pathology [4].

Ventricular remodelling refers to alterations to the geometry, structure and function of the heart in response to changes in loading conditions. The natural history of LV dimensions and function in the setting of chronic valvular RHD over different time points has not been explored. This has clinical implications on the frequency of follow-up of patients in remote settings given the likelihood of worsening LV remodelling over time. There is evidence to support the negative impact of LV dilatation and dysfunction in mitral valve disease potentially guiding the timing of surgical intervention, as late cardiac surgery may not lead to LV recovery [1, 5, 6, 7].

In Western Australia (WA), we have observed that LV dilatation and systolic function remained stable in both mild and moderate severity RHD and that some pediatric patients with severe RHD showed stability in the degree of valvular regurgitation and LV dilatation, therefore avoiding surgery. We thus sought to characterize the natural history of LV remodelling over time in different RHD severity groups. We hypothesized that LV remodelling in mild RHD would remain stable, whilst moderate and severe RHD groups would develop progressive LV dilatation and dysfunction over time. Secondary objectives included comparing LV parameters at diagnosis in the above RHD patients to (a) healthy controls and (b) those referred for early surgical intervention.

## Methods

### Study setting and design

A retrospective review of 146 pediatric patients (≤ 16 years at diagnosis) meeting RHD diagnostic criteria between January 1987 to December 2020 was performed at Perth Children’s Hospital (formally Princess Margaret Hospital), the only tertiary paediatric hospital in WA. Cases were identified from the Cardiology echocardiographic database (Synapse™ Cardiovascular Client V4.0.4, Fujifilm Medical Systems USA) with validation of cases using the institutional Cardiology electronic clinical database (Cardiobase™, Version 8.1.44.10, Derby UK).

We analysed a subset of these patients with three or more echocardiograms, at least 12 months follow-up duration and no surgical intervention during this time, hereafter defined as the ‘natural history cohort’. Echocardiograms at diagnosis (T1), follow-up at 12 months (T2) and, where possible, their most recent follow-up between two and five years after diagnosis (T3or *outcome echocardiogram*) were selected to provide consistent and comparable timepoints. If echocardiogram quality was not satisfactory the next available echocardiogram within three months was used. Stratification into groups (mild, moderate or severe RHD) was based on diagnostic echocardiogram according to Australian RHD guideline definitions (Table 1) [4]. We excluded patients with concomitant significant unrepaired congenital heart disease and those without three echocardiograms or a diagnostic (initial) echocardiogram available. ARF recurrence was documented in clinical descriptions of the natural history cohort.

**Table 1:** Australian 2020 RHD guidelines definition criteria for a risk stratification of RHD [4]

*Diagnosis*	*Definition*
*Mild RHD*	Echocardiogram showing: Mild regurgitation or mild stenosis of a single valve OR Atrioventricular conduction abnormality on ECG during ARF episode
*Moderate RHD*	Echocardiogram showing: Moderate regurgitation or moderate stenosis of a single valve OR Combined mild regurgitation and/or mild stenosis of one or more valves
*Severe RHD*	Echocardiogram showing: Severe regurgitation or severe stenosis of any valve OR Combined moderate regurgitation and/or moderate stenosis of one or more valves OR Past or impending valve repair or prosthetic valve replacement

### Definitions

For the purpose of the study, left ventricular remodelling was defined as the change in volume and function over time, secondary to the effects of valvular regurgitation on the left heart. Postcodes at the time of first RHD diagnosis were used to categorize remoteness, as per Accessibility/Remoteness Index of Australia (ARIA 2011) (produced by the Australian Bureau of Statistics), which indicates distance from that postcode and accessibility to service centres [8]. Ethnicity was defined by patient self-identification recorded in hospital administrative records.

### Comparison groups

We included fifty age- and sex-matched healthy children with normal echocardiograms and no underlying cardiac conditions that were randomly selected for comparison to LV parameters. Only initial echocardiograms at diagnosis were compared as matched controls only have a single echocardiogram available. We included a comparision surgical RHD patients with single pre-operative echocardiograms (n = 17) to baseline measures of the natural history cohort for completeness (supplementary Table 1).

### Echocardiographic measurements

Transthoracic echocardiograms (TTE) were reviewed in accordance with American Society of Echocardiography [9] and World Heart Federation criteria for RHD guidelines [3]. These were on either Philips (IE33; Philips Ultrasound, Bothell, WA USA) or GE (Vivid Q or Vivid IQ, GE Healthcare, Chicago, USA) echocardiography machines with sector phased array broadband transducers X5-1 MHz or the S8-3 MHz (Philips Ultrasound, Bothell, WA USA). Where limitations in grading of valvular regurgitation occurred then clinician expert opinion was used to guide diagnosis of valvular regurgitation in RHD [3]. Where feasible, measurements were extracted from reports at the time of echocardiogram. If not available, measures were retaken by investigators. Twenty randomly selected echocardiograms were re-analysed by an echocardiographer blinded to recorded measurements to assess inter-observer variability.

Parameters collected included mitral valve annular dimension (cm), left atrial (LA) area (cm^2^), LV end-diastolic dimension (M-mode), LV end-systolic dimension (M-mode), septal and posterior wall thickness in diastole, LV apical 4-chamber end-diastolic and end-systolic indexed volumes, LV apical 4-chamber end-systolic area. LV end-diastolic volumes (EDV) and end-systolic volumes (ESV) were quantified using Simpson’s biplane method and indexed to body surface area. Boston Z-scores, accessed January 2021, were used where required [10].

### Statistical analysis

Descriptive statistics including counts, percentages, means or standard deviations (median and interquartile range (IQR) where appropriate) were used to describe RHD patients at diagnosis. Linear mixed effect models adjusted for sex, ethnicity and remoteness were used to assess associations between disease severity and timepoint in the natural history cohort. Linear regression was used for comparisons of the baseline echocardiogram results of the natural history cohort with those of matched controls and early surgery patients. Unless otherwise noted, mean estimated differences and 95% confidence intervals (CIs) with corresponding p-values are presented. Significance was determined at p < 0.05 (2-tailed) with Tukey’s adjustments for multiple comparisons. Statistical analysis was performed using R version 4.1.0 (R Foundation for Statistical Computing, Vienna, Austria) [11].

### Ethics approval

*Ethics and waiver of consent was approved by the Child and Adolescent Health Service Human Research Ethics Committee (Research Governance Service #0000003471). All methods were carried out in accordance with relevant guidelines and regulations. All experimental protocols were approved by the named institutional committee. A waiver of consent was obtained for all patients as per the Child and Adolescent Health Service Human Research Ethics Committee.*

## Results

### Descriptive characteristics of RHD natural history cohort

Of the 221 patients with RHD on our databases we found 146 patients with adequate echocardiograms (in both quality and number) available for analysis. 90% of this natural disease cohort were aged 5–14 years at diagnosis (median age 10 years (IQR 4)) and 51% were male. Patients largely identified as Aboriginal or Torres Strait Islander (91%). Over half (57%) were from *very remote areas* (Table 2). Average duration of follow-up was 4.6 years from the initial diagnosis. Median ages at echocardiograms were 10 years (IQR 4 years) at T1, 12 years (IQR 3 years) at T2, and 15 years (IQR 5 years) at *outcome* ranging in patients aged between 3 and 18 years of age.

### Severity of valvular disease

Over half (n = 85; 58%) of the natural history cohort, fulfilled criteria for mild disease, 33 (23%) for moderate and 28 (19%) for severe disease. Mitral regurgitation (MR) occurred in 100 (68%) patients, aortic regurgitation (AR) in 16 patients (11%) and combined valve involvement in 31 patients (21%). Five patients had isolated AR. No patients presented with mitral or aortic stenosis.

Hospital admission was required in 68 (46.6%) patients for management of carditis with or without heart failure. Ten patients (7%) from the severe group progressed to surgery beyond the follow-up period (> 2 years). Valvular regurgitation worsened in the context of recurrence of ARF in six (7%) patients in the mild group and one (3%) in the moderate group. In the severe group, 15 patients (53%) had stable or improved valvular regurgitation over time. No deaths occurred within the paediatric service follow-up.

Recurrence of ARF was documented in 21 patients (14%), including 10 of the 85 patients with mild disease (12%), one of 33 with moderate disease (3%) and 10 of the 28 with severe disease (36%).

**Table 2:** Demographic details of Rheumatic Heart Disease natural history cohort (2003–2020)

		*Mild*	*Moderate*	*Severe*	*TOTAL*
*Patient*		85 (58)	33 (23)	28 (19)	146 (100)
*Age group*	0–4	4 (36)	3 (27)	1 (9)	8 (5)
	5–14	78 (46)	28 (17)	24 (14)	130 (90)
	> 14	3 (19)	2 (12)	3 (19)	8 (5)
*Sex*	Male	44 (52)	17 (52)	14 (50)	75 (51)
*Population group*	Aboriginal/ Torres Strait Islander	81 (95)	27 (82)	25 (89)	133 (91)
	Other	4 (5)	6 (18)	3 (11)	13 (9)
*ARIA*	Major cities	21 (25)	9 (27)	8 (29)	38 (27)
	Inner regional	3 (4)	3 (9)	3 (11)	9 (6)
	Outer regional	2 (2)	4 (12)	2 (7)	8 (5)
	Remote	7 (8)	1 (3)	0 (0)	8 (5)
	Very remote	52 (61)	16 (48)	15 (54)	83 (57)

*N (%); ARIA = Accessibility and Remoteness Index, a score that indicates distance of geographical location and therefore accessibility to service centres*

### Changes over time according to RHD severity

**Fig. 1:**
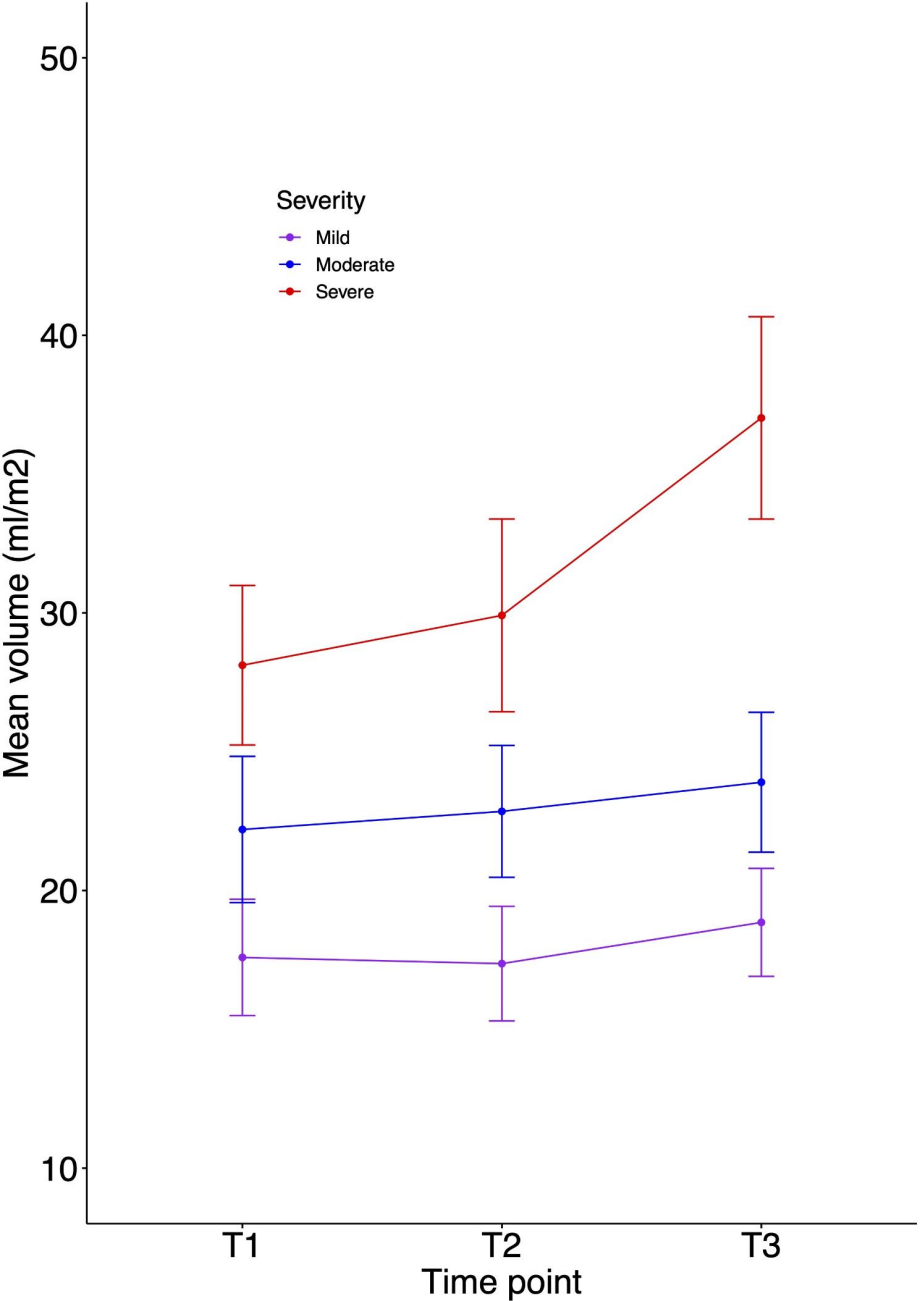
End-systolic indexed volumes in paediatric RHD over follow up time points

**Fig. 2:**
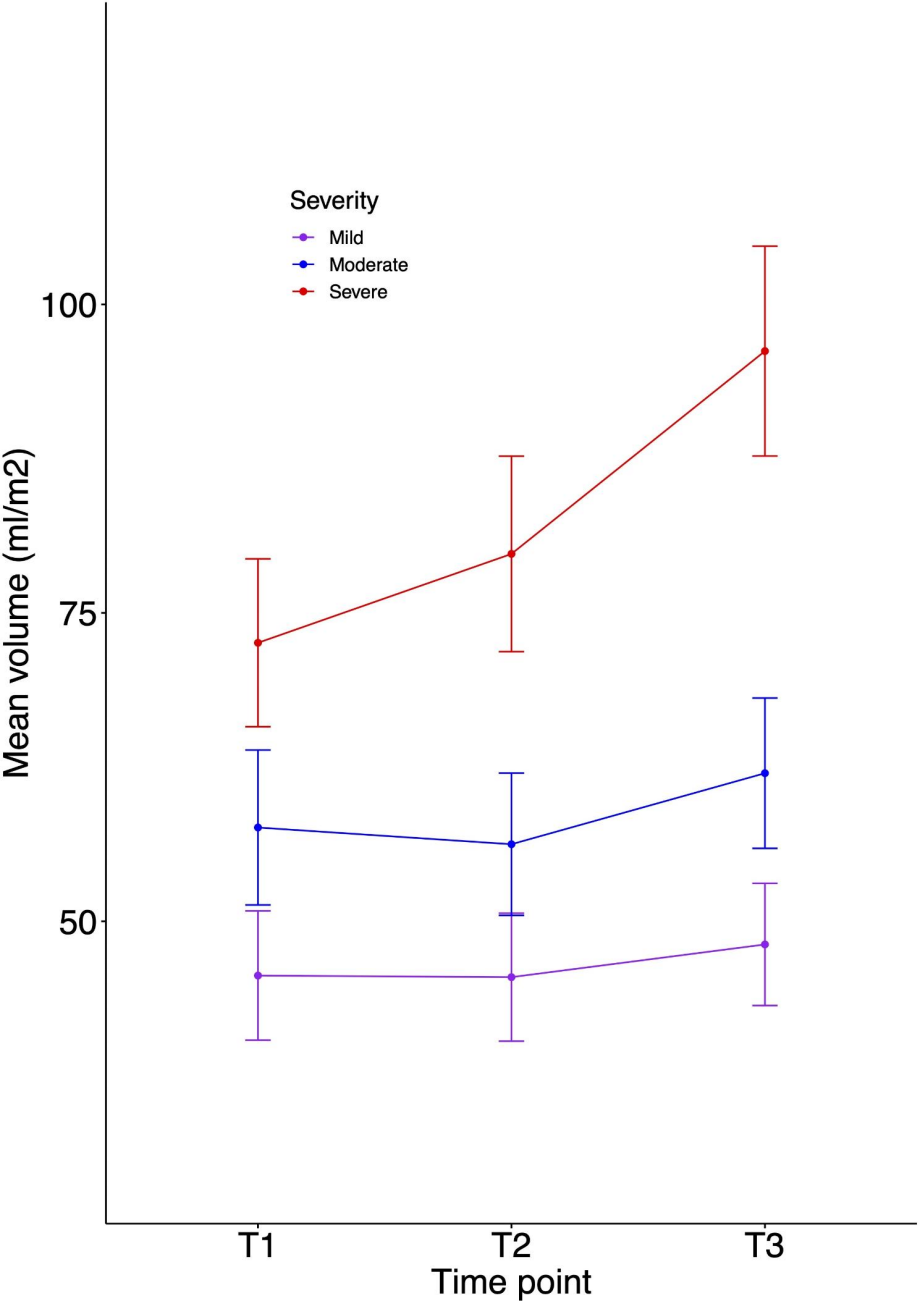
End-diastolic indexed volumes in paediatric RHD over follow up time points

In the mild group, indexed volumes were unchanged (supplementary Tables 2 and 3) over time. Similarly, in the moderate group, LV indexed volume measurements did not change significantly across timepoints. M-mode LV parameters of LVEDD (mean difference z-score 1.10 (95% CI 0.34–1.86); p < 0.001) and LVESD (0.93 (0.23–1.63); p < 0.001) changed marginally over time. The severe group, however, had a significant increase in indexed EDV at T2 (23.64 ml/m^2^ (10.3036.99); p < 0.001) and *outcome* (16.44ml/m^2^ (2.33–30.54) p = 0.01) with corresponding changes in indexed ESV measurements. The trajectory of LV volumetry in all severity groups is highlighted in Fig. 1 (ESV) and Fig. 2 (EDV). Additionally, M-mode LVEDD z-scores increased between baseline and *outcome* (1.10 (0.09–2.09), p = 0.02), as did the LA area (4.77cm^2^ (1.56–7.98), p = 0.001) and MV annulus dimension (0.38 (0.06–0.7), p = 0.01). LV ejection fraction remained stable across all groups over time (supplementary Table 3).

### Comparisons between severity groups

At baseline, the mild RHD group had significantly smaller measures of LVEDD, LVESD, LA area and indexed EDV than moderate and severe groups (supplementary Table 3). At baseline, the severe RHD group had significantly larger volumes compared to mild RHD in all measures; including EDV (26.97ml/m^2^ (16.20-37.74); p < 0.001) and ESV (10.53 ml/m^2^ (5.75–15.31); p < 0.001). The mean difference further increased between groups at *outcome* for EDV (48.09 ml/m^2^ (34.79–61.40); p < 0.001) and ESV (18.17 ml/m^2^(12-32-24.03); p < 0.001). Similar changes are seen between the severe and moderate group (supplementary Table 3). Smaller differences between moderate and mild severity groups are seen at baseline with EDV (12.00 ml/m^2^ (2.16–21.84); p = 0.01) and ESV (4.61 ml/m^2^(0.20–9.02); p = 0.03), which at follow up time points remain significant with ESV 5.05 ml/m^2^ (0.97–9.13); p < 0.001] and EDV 13.88 ml/m^2^(4.62–23.14); p < 0.001 (Figs. 1 and 2).

### Comparisons to healthy controls and early surgical cohort

The mild RHD group showed no difference to the healthy controls for all T1 LV measures (supplementary Table 1). The moderate group was significantly different to the control group for LVEDD (mean difference z-score 1.47 (0.27–2.66); p < 0.01) but other measures were comparable. All parameters in the severe group were significantly different from controls with the exceptions being LA area and MV annulus dimension.

The early surgical cohort showed significantly larger left ventricular volumes and two dimensional measurements across almost all measures compared to mild, moderate and severe patients at baseline. Left ventricular systolic function was preserved with no significant differences between controls and severity groups (supplementary Table 1).

### Inter-observer variability

Twenty echocardiographic studies were randomly selected to assess for inter-observer variability. There was good overall agreement with acceptable reproducibility for randomly selected parameters when measures were repeated by a sonographer blinded to the prior results. The correlation coefficients ranged between 0.81 and 0.92 and intraclass correlations 0.59–0.92 for various measurements, outlined in the (supplementary Table 4).

## Discussion

We present a large paediatric RHD cohort from a statewide Australian clinical service. To our knowledge, this is the first longitudinal echocardiographic study in a cohort of patients reporting the trend of LV remodelling over the course of childhood RHD. We characterized the chronic effects of LV volume loading from valvular pathology and found no significant progression of LV parameters and function in patients with mild and moderate RHD. The patients with severe RHD already demonstrated considerable LV dilatation at diagnosis. Our data suggests that in mild and moderate RHD, the current one to two-yearly follow-up echocardiograms are reasonable and may be reviewed knowing that progression of LV dilatation and dysfunction is unlikely.

Anticipating progression of RHD in Australia is critical as many patients with RHD live in rural and remote regions where echocardiographic follow up may be more infrequent [1, 3, 12]. A prior study suggested that mild RHD can progress to severe disease [13], though reassuringly we did not observe this deterioration in our patients with mild to moderate disease. In our experience, worsening valvular regurgitation in the absence of RHD recurrence is uncommon over the course of childhood [14]. This suggests that in milder RHD, cardiology follow up over the remainder of childhood may be undertaken with confidence that progression of LV volume measures, and therefore the need for intervention, would be unlikely. Our findings support the current RHD guidelines of one to two yearly follow-up in the absence of interim ARF recurrence [4]. This is highly relevant in WA, where the majority of patients with ARF and RHD come from rural and remote settings and access to tertiary Cardiology services are limited. Distance to hospital is certainly a factor, amongst many, that is considered in surgical decision making in severe RHD. If we can confidently predict the medium to long-term outlook for this cohort then we can balance safety and convenience when reviewing the timing of follow-up.

Patients with severe RHD were found to have significant LV remodelling with left chamber dilatation at diagnosis; with preload and stroke volume increases in the presence of severe valvular regurgitation. Similar to Gaasch et al., chronic valvular regurgitation in our cohort lead to further increases in LV dimensions and volumes over time, likely a compensatory mechanism to deliver a larger stroke volume owing to LV systolic unloading [15]. It is reassuring that despite these compensatory changes, the LV systolic function remained preserved across all severity groups over time, reflecting the ability for the compliant LV to adapt to chronic volume loading conditions.

We found that patients who underwent early surgical intervention had significantly increased LV parameters compared to all severity groups in the natural history cohort, including patients in the severe RHD group who were managed medically. The decision of referral for surgical intervention has historically been difficult in the RHD cohort. In our institution, multiple clinical and non-clinical factors are considered, including symptomatic heart failure, significant valvular regurgitation, increasing LV dilatation and/or dysfunction over time, reduced functional capacity, rurality and predicted compliance to follow up and secondary prophylaxis [4, 5, 16, 17]. Where possible, a ‘watchful waiting’ approach is adopted in asymptomatic patients with moderate or more RHD in the absence of worsening LV parameters [18]. However, considerable morbidity in severe RHD is reflected in this cohort, with 10 patients requiring surgery beyond our study period of interest.

The strength of our study was that it includes robust echocardiogram measures of a relatively large paediatric cohort of RHD sufferers. Additionally, the ability to follow up these patients over childhood with comparable echocardiograms, validated by technicians, within the same centre is another strength. There were several limitations to this study owing to the retrospective study design. As our primary aim was to study the natural history of RHD severity over time, the selected cohort was limited to those who had appropriate follow-up time points and adequate quality of echocardiograms without interim interventions, thus limiting patient numbers. Even so, there continues to be extensive issues with the timing of echocardiograms in this cohort, which becomes problematic in the design and analysis of the echocardiograms. Most notably, our T3 or outcome measure is highly variable in timing and has a relatively wide follow up time from diagnosis. Our T3 measure does not always represent a point in time but more reflects the last known echocardiogram measures for that patient. This reflects the real world issues with a complex disease and our study sometimes suffers in rigor as a result of this. Additionally, the *natural history* cohort was only half of all tertiary referred RHD patients given limitations in ascertainment of echocardiograms at necessary timepoints, although remains similar representative of the RHD cohort as a whole with respect to age, severity and ethnicity but calendar period.

## Conclusion

Rheumatic heart disease remains a significant health burden in Australia and negatively impacts quality of life, morbidity and mortality. Understanding the natural history of LV remodelling provides important insights into how chronic valvular RHD affects LV geometry and function in paediatric patients. Our findings may guide clinicians in deciding the frequency and timing of follow-up and knowledge of the expected trajectory of disease may be of clinical utility during further reiterations of the Australia and New Zealand RHD Guidelines^4^. Further longitudinal studies following this paediatric cohort into adulthood are warranted to assess how chronic LV volume loading affects their disease trajectory and long-term outcomes.

### Electronic supplementary material

Below is the link to the electronic supplementary material.

Supplementary Material 1

## Data Availability

The analysed data underlying this article are available in the article and in its online supplementary material. The individual data underlying this article cannot be publicly shared due to identifying factors that would potentially breach the privacy of individuals.
