# Supplementary material for: Left ventricular remodelling in rheumatic heart disease – trends over time and implications for follow-up in childhood
Source: BMC Cardiovasc Disord. 2023 Sep 15;23:462. doi: 10.1186/s12872-023-03497-0 (PMC10503178; doi:10.1186/s12872-023-03497-0)
Supplement: Supplementary file 1 — Supplementary Material 1 [file 12872_2023_3497_MOESM1_ESM.docx]

**Appendices**

#### *Supplementary Table 1:* *Adjusted mean differences (and 95% CIs) for baseline (T1) LV parameters using linear regression show significantly remodelled hearts in more severe disease and surgical patients.*

| **Measure** | **Contrasting measures** | **Mean estimate difference** | **Lower CI** | **Upper CI** | **P-value** |
| --- | --- | --- | --- | --- | --- |
| *LVEDD Z score* | Control - Mild | 0.12 | -1.05 | 1.29 | 1.00 |
|  | Control - Moderate | -1.47 | -2.66 | -0.27 | 0.01 |
|  | Control - Severe | -2.91 | -4.17 | -1.66 | <0.001 |
|  | Control - Surgical | -5.03 | -6.21 | -3.86 | <0.001 |
|  | Mild - Moderate | -1.59 | -2.37 | -0.81 | <0.001 |
|  | Mild - Severe | -3.04 | -3.88 | -2.20 | <0.001 |
|  | Mild - Surgical | -5.16 | -6.60 | -3.71 | <0.001 |
|  | Moderate - Severe | -1.45 | -2.42 | -0.47 | <0.001 |
|  | Moderate - Surgical | -3.56 | -5.03 | -2.10 | <0.001 |
|  | Severe - Surgical | -2.12 | -3.62 | -0.61 | <0.001 |
| *LVESD Z score* | Control - Mild | 0.04 | -1.06 | 1.14 | 1.00 |
|  | Control - Moderate | -1.13 | -2.25 | -0.01 | 0.05 |
|  | Control - Severe | -1.93 | -3.11 | -0.75 | <0.001 |
|  | Control - Surgical | -4.21 | -5.31 | -3.10 | <0.001 |
|  | Mild - Moderate | -1.17 | -1.91 | -0.44 | <0.001 |
|  | Mild - Severe | -1.97 | -2.76 | -1.18 | <0.001 |
|  | Mild - Surgical | -4.25 | -5.61 | -2.89 | <0.001 |
|  | Moderate - Severe | -0.80 | -1.71 | 0.12 | 0.12 |
|  | Moderate - Surgical | -3.08 | -4.46 | -1.70 | <0.001 |
|  | Severe - Surgical | -2.28 | -3.70 | -0.86 | <0.001 |
| *LA area 4ch (cm2)* | Control - Mild | 1.35 | -3.44 | 6.14 | 0.94 |
|  | Control - Moderate | -1.99 | -6.80 | 2.82 | 0.79 |
|  | Control - Severe | -5.27 | -10.36 | -0.18 | 0.04 |
|  | Control - Surgical | -10.59 | -15.37 | -5.81 | <0.001 |
|  | Mild - Moderate | -3.34 | -6.40 | -0.28 | 0.02 |
|  | Mild - Severe | -6.62 | -9.84 | -3.39 | <0.001 |
|  | Mild - Surgical | -11.94 | -17.80 | -6.08 | <0.001 |
|  | Moderate - Severe | -3.28 | -7.07 | 0.51 | 0.12 |
|  | Moderate - Surgical | -8.60 | -14.46 | -2.74 | <0.001 |
|  | Severe - Surgical | -5.32 | -11.43 | 0.78 | 0.12 |
| *MV annulus Z score* | Control - Mild | 0.06 | -0.28 | 0.40 | 0.99 |
|  | Control - Moderate | -0.09 | -0.43 | 0.25 | 0.95 |
|  | Control - Severe | -0.16 | -0.52 | 0.20 | 0.75 |
|  | Control - Surgical | -0.64 | -0.96 | -0.32 | <0.001 |
|  | Mild - Moderate | -0.15 | -0.36 | 0.07 | 0.34 |
|  | Mild - Severe | -0.21 | -0.44 | 0.01 | 0.08 |
|  | Mild - Surgical | -0.70 | -1.10 | -0.30 | <0.001 |
|  | Moderate - Severe | -0.07 | -0.34 | 0.20 | 0.96 |
|  | Moderate - Surgical | -0.55 | -0.95 | -0.15 | <0.001 |
|  | Severe - Surgical | -0.48 | -0.90 | -0.07 | 0.01 |
| *IVS Z score* | Control - Mild | 0.33 | -0.66 | 1.33 | 0.89 |
|  | Control - Moderate | 0.01 | -1.01 | 1.02 | 1.00 |
|  | Control - Severe | -0.61 | -1.67 | 0.45 | 0.51 |
|  | Control - Surgical | -0.69 | -1.69 | 0.30 | 0.31 |
|  | Mild - Moderate | -0.33 | -0.99 | 0.34 | 0.65 |
|  | Mild - Severe | -0.94 | -1.66 | -0.23 | <0.001 |
|  | Mild - Surgical | -1.03 | -2.26 | 0.20 | 0.15 |
|  | Moderate - Severe | -0.61 | -1.44 | 0.21 | 0.25 |
|  | Moderate - Surgical | -0.70 | -1.94 | 0.54 | 0.53 |
|  | Severe - Surgical | -0.09 | -1.37 | 1.19 | 1.00 |
| *LVPWD Z score* | Control - Mild | 0.11 | -0.96 | 1.18 | 1.00 |
|  | Control - Moderate | 0.20 | -0.89 | 1.29 | 0.99 |
|  | Control - Severe | -0.28 | -1.42 | 0.86 | 0.96 |
|  | Control - Surgical | -0.96 | -2.03 | 0.11 | 0.10 |
|  | Mild - Moderate | 0.09 | -0.63 | 0.80 | 1.00 |
|  | Mild - Severe | -0.39 | -1.16 | 0.38 | 0.62 |
|  | Mild - Surgical | -1.07 | -2.39 | 0.25 | 0.17 |
|  | Moderate - Severe | -0.48 | -1.36 | 0.41 | 0.58 |
|  | Moderate - Surgical | -1.16 | -2.49 | 0.18 | 0.12 |
|  | Severe - Surgical | -0.68 | -2.05 | 0.70 | 0.65 |
| *LV 4ch EF (%)* | Control - Mild | 3.76 | -1.17 | 8.69 | 0.22 |
|  | Control - Moderate | 3.22 | -1.73 | 8.16 | 0.38 |
|  | Control - Severe | 2.89 | -2.34 | 8.13 | 0.55 |
|  | Control - Surgical | 1.82 | -2.99 | 6.63 | 0.84 |
|  | Mild - Moderate | -0.54 | -3.69 | 2.60 | 0.99 |
|  | Mild - Severe | -0.87 | -4.18 | 2.45 | 0.95 |
|  | Mild - Surgical | -1.94 | -7.88 | 4.00 | 0.90 |
|  | Moderate - Severe | -0.32 | -4.22 | 3.57 | 1.00 |
|  | Moderate - Surgical | -1.40 | -7.33 | 4.54 | 0.97 |
|  | Severe - Surgical | -1.07 | -7.27 | 5.12 | 0.99 |
| *LV EDVi 4ch (ml/m2)* | Control - Mild | 13.36 | -1.38 | 28.11 | 0.10 |
|  | Control - Moderate | 1.54 | -13.52 | 16.61 | 1.00 |
|  | Control - Severe | -23.50 | -39.27 | -7.73 | <0.001 |
|  | Control - Surgical | -52.86 | -66.45 | -39.28 | <0.001 |
|  | Mild - Moderate | -11.82 | -21.67 | -1.97 | 0.01 |
|  | Mild - Severe | -36.86 | -47.44 | -26.28 | <0.001 |
|  | Mild - Surgical | -66.23 | -83.46 | -49.00 | <0.001 |
|  | Moderate - Severe | -25.04 | -37.32 | -12.76 | <0.001 |
|  | Moderate - Surgical | -54.40 | -71.88 | -36.93 | <0.001 |
|  | Severe - Surgical | -29.37 | -47.38 | -11.35 | <0.001 |
| *LV ESVi 4ch (ml/m2)* | Control - Mild | 3.11 | -3.16 | 9.38 | 0.65 |
|  | Control - Moderate | -0.97 | -7.37 | 5.44 | 0.99 |
|  | Control - Severe | -10.15 | -16.85 | -3.44 | <0.001 |
|  | Control - Surgical | -19.92 | -25.70 | -14.14 | <0.001 |
|  | Mild - Moderate | -4.08 | -8.27 | 0.11 | 0.06 |
|  | Mild - Severe | -13.26 | -17.76 | -8.76 | <0.001 |
|  | Mild - Surgical | -23.03 | -30.36 | -15.71 | <0.001 |
|  | Moderate - Severe | -9.18 | -14.40 | -3.96 | <0.001 |
|  | Moderate - Surgical | -18.95 | -26.38 | -11.52 | <0.001 |
|  | Severe - Surgical | -9.77 | -17.43 | -2.11 | 0.01 |

Left atrium (LA), four chamber (4ch) posterior long axis view (PLAX), mitral valve (MV), anterior (Ant.), interventricular septum (IVS), left ventricular end diastolic diameter in posterior long axis view (LVEDD), left ventricular posterior wall diameter (LVPWD), left ventricular end systolic volume in posterior long axis view (LVESD), left ventricular (LV), end diastolic volume indexed to BSA (EDVi), end systolic volume indexed to BSA (ESVi), ejection fraction (EF), confidence interval (CI), T1 = timepoint 1, T2 = timepoint 2, T3 = timepoint 3.

#### *Supplementary Table 2:* *Adjusted mean differences (and 95% CIs) for LV parameters based on linear mixed effect models comparing RHD severity and timepoint showing that mild and moderate disease are often similar and do not progress over time.*

| **Measure** | **Contrasting measures** | **Adjusted mean difference (95% CI)** | **Lower CI** | **Upper CI** | **P-value** |
| --- | --- | --- | --- | --- | --- |
| *LVEDD Z score* | Mild T1 - Mild T2 | -0.04 | -0.51 | 0.43 | 1.00 |
|  | Mild T1 - Mild T3 | 0.34 | -0.11 | 0.79 | 0.32 |
|  | Mild T1 - Moderate T1 | -1.50 | -2.26 | -0.74 | <0.001 |
|  | Mild T1 - Severe T1 | -2.42 | -3.25 | -1.59 | <0.001 |
|  | Mild T2 - Mild T3 | 0.38 | -0.05 | 0.82 | 0.14 |
|  | Mild T2 - Moderate T2 | -0.76 | -1.45 | -0.08 | 0.02 |
|  | Mild T2 - Severe T2 | -2.49 | -3.46 | -1.51 | <0.001 |
|  | Mild T3 - Moderate T3 | -0.74 | -1.45 | -0.03 | 0.04 |
|  | Mild T3 - Severe T3 | -3.86 | -4.91 | -2.82 | <0.001 |
|  | Moderate T1 - Moderate T2 | 0.70 | -0.03 | 1.42 | 0.07 |
|  | Moderate T1 - Moderate T3 | 1.10 | 0.34 | 1.86 | <0.001 |
|  | Moderate T1 - Severe T1 | -0.92 | -1.84 | 0.00 | 0.05 |
|  | Moderate T2 - Moderate T3 | 0.41 | -0.28 | 1.10 | 0.65 |
|  | Moderate T2 - Severe T2 | -1.72 | -2.72 | -0.73 | <0.001 |
|  | Moderate T3 - Severe T3 | -3.12 | -4.23 | -2.02 | <0.001 |
|  | Severe T1 - Severe T2 | -0.11 | -1.07 | 0.86 | 1.00 |
|  | Severe T1 - Severe T3 | -1.10 | -2.14 | -0.07 | 0.03 |
|  | Severe T2 - Severe T3 | -1.00 | -2.09 | 0.09 | 0.10 |
| *LVESD Z score* | Mild T1 - Mild T2 | -0.03 | -0.47 | 0.40 | 1.00 |
|  | Mild T1 - Mild T3 | 0.15 | -0.27 | 0.56 | 0.97 |
|  | Mild T1 - Moderate T1 | -1.04 | -1.73 | -0.35 | <0.001 |
|  | Mild T1 - Severe T1 | -1.51 | -2.27 | -0.76 | <0.001 |
|  | Mild T2 - Mild T3 | 0.18 | -0.22 | 0.58 | 0.89 |
|  | Mild T2 - Moderate T2 | -0.54 | -1.17 | 0.08 | 0.14 |
|  | Mild T2 - Severe T2 | -1.93 | -2.81 | -1.04 | <0.001 |
|  | Mild T3 - Moderate T3 | -0.26 | -0.90 | 0.39 | 0.95 |
|  | Mild T3 - Severe T3 | -2.39 | -3.35 | -1.44 | <0.001 |
|  | Moderate T1 - Moderate T2 | 0.47 | -0.20 | 1.13 | 0.42 |
|  | Moderate T1 - Moderate T3 | 0.93 | 0.23 | 1.63 | <0.001 |
|  | Moderate T1 - Severe T1 | -0.47 | -1.31 | 0.37 | 0.72 |
|  | Moderate T2 - Moderate T3 | 0.47 | -0.17 | 1.10 | 0.34 |
|  | Moderate T2 - Severe T2 | -1.38 | -2.30 | -0.47 | <0.001 |
|  | Moderate T3 - Severe T3 | -2.14 | -3.15 | -1.13 | 0.00 |
|  | Severe T1 - Severe T2 | -0.45 | -1.34 | 0.44 | 0.82 |
|  | Severe T1 - Severe T3 | -0.73 | -1.69 | 0.22 | 0.29 |
|  | Severe T2 - Severe T3 | -0.29 | -1.29 | 0.72 | 0.99 |
| *LA area 4ch (cm2)* | Mild T1 - Mild T2 | -0.34 | -1.81 | 1.13 | 1.00 |
|  | Mild T1 - Mild T3 | -2.17 | -3.60 | -0.74 | 0.00 |
|  | Mild T1 - Moderate T1 | -3.17 | -5.56 | -0.78 | <0.001 |
|  | Mild T1 - Severe T1 | -4.90 | -7.53 | -2.28 | <0.001 |
|  | Mild T2 - Mild T3 | -1.83 | -3.25 | -0.41 | <0.001 |
|  | Mild T2 - Moderate T2 | -2.75 | -5.03 | -0.48 | 0.01 |
|  | Mild T2 - Severe T2 | -6.65 | -9.79 | -3.52 | <0.001 |
|  | Mild T3 - Moderate T3 | -3.51 | -5.87 | -1.14 | <0.001 |
|  | Mild T3 - Severe T3 | -7.50 | -10.84 | -4.16 | <0.001 |
|  | Moderate T1 - Moderate T2 | 0.07 | -2.27 | 2.42 | 1.00 |
|  | Moderate T1 - Moderate T3 | -2.51 | -4.96 | -0.05 | 0.04 |
|  | Moderate T1 - Severe T1 | -1.73 | -4.64 | 1.18 | 0.64 |
|  | Moderate T2 - Moderate T3 | -2.58 | -4.88 | -0.28 | 0.02 |
|  | Moderate T2 - Severe T2 | -3.90 | -7.14 | -0.66 | 0.01 |
|  | Moderate T3 - Severe T3 | -4.00 | -7.53 | -0.46 | 0.01 |
|  | Severe T1 - Severe T2 | -2.10 | -5.13 | 0.94 | 0.44 |
|  | Severe T1 - Severe T3 | -4.77 | -7.98 | -1.56 | <0.001 |
|  | Severe T2 - Severe T3 | -2.68 | -6.09 | 0.74 | 0.26 |
| *MV annulus Z score* | Mild T1 - Mild T2 | -0.04 | -0.19 | 0.11 | 1.00 |
|  | Mild T1 - Mild T3 | -0.26 | -0.40 | -0.11 | <0.001 |
|  | Mild T1 - Moderate T1 | -0.15 | -0.38 | 0.08 | 0.55 |
|  | Mild T1 - Severe T1 | -0.20 | -0.45 | 0.05 | 0.26 |
|  | Mild T2 - Mild T3 | -0.22 | -0.36 | -0.08 | <0.001 |
|  | Mild T2 - Moderate T2 | -0.11 | -0.33 | 0.10 | 0.80 |
|  | Mild T2 - Severe T2 | -0.45 | -0.74 | -0.15 | <0.001 |
|  | Mild T3 - Moderate T3 | -0.11 | -0.34 | 0.12 | 0.85 |
|  | Mild T3 - Severe T3 | -0.32 | -0.64 | 0.00 | 0.05 |
|  | Moderate T1 - Moderate T2 | 0.00 | -0.24 | 0.23 | 1.00 |
|  | Moderate T1 - Moderate T3 | -0.22 | -0.47 | 0.02 | 0.12 |
|  | Moderate T1 - Severe T1 | -0.05 | -0.33 | 0.24 | 1.00 |
|  | Moderate T2 - Moderate T3 | -0.22 | -0.45 | 0.01 | 0.08 |
|  | Moderate T2 - Severe T2 | -0.34 | -0.65 | -0.02 | 0.03 |
|  | Moderate T3 - Severe T3 | -0.21 | -0.55 | 0.14 | 0.62 |
|  | Severe T1 - Severe T2 | -0.29 | -0.59 | 0.02 | 0.08 |
|  | Severe T1 - Severe T3 | -0.38 | -0.70 | -0.06 | 0.01 |
|  | Severe T2 - Severe T3 | -0.09 | -0.44 | 0.25 | 1.00 |
| *IVS Z score* | Mild T1 - Mild T2 | -0.42 | -0.86 | 0.02 | 0.08 |
|  | Mild T1 - Mild T3 | -0.43 | -0.85 | -0.01 | 0.04 |
|  | Mild T1 - Moderate T1 | -0.40 | -1.04 | 0.24 | 0.58 |
|  | Mild T1 - Severe T1 | -0.88 | -1.57 | -0.19 | <0.001 |
|  | Mild T2 - Mild T3 | -0.01 | -0.42 | 0.40 | 1.00 |
|  | Mild T2 - Moderate T2 | -0.26 | -0.83 | 0.30 | 0.87 |
|  | Mild T2 - Severe T2 | -0.76 | -1.56 | 0.04 | 0.08 |
|  | Mild T3 - Moderate T3 | -0.08 | -0.67 | 0.51 | 1.00 |
|  | Mild T3 - Severe T3 | -0.05 | -0.88 | 0.78 | 1.00 |
|  | Moderate T1 - Moderate T2 | -0.29 | -0.95 | 0.38 | 0.92 |
|  | Moderate T1 - Moderate T3 | -0.11 | -0.81 | 0.58 | 1.00 |
|  | Moderate T1 - Severe T1 | -0.48 | -1.27 | 0.31 | 0.61 |
|  | Moderate T2 - Moderate T3 | 0.17 | -0.46 | 0.81 | 1.00 |
|  | Moderate T2 - Severe T2 | -0.49 | -1.34 | 0.36 | 0.67 |
|  | Moderate T3 - Severe T3 | 0.03 | -0.89 | 0.95 | 1.00 |
|  | Severe T1 - Severe T2 | -0.30 | -1.18 | 0.58 | 0.98 |
|  | Severe T1 - Severe T3 | 0.40 | -0.52 | 1.32 | 0.91 |
|  | Severe T2 - Severe T3 | 0.70 | -0.29 | 1.68 | 0.41 |
| *LVPWD Z score* | Mild T1 - Mild T2 | -0.36 | -0.86 | 0.14 | 0.38 |
|  | Mild T1 - Mild T3 | -0.52 | -1.00 | -0.04 | 0.02 |
|  | Mild T1 - Moderate T1 | 0.13 | -0.61 | 0.87 | 1.00 |
|  | Mild T1 - Severe T1 | -0.36 | -1.16 | 0.43 | 0.89 |
|  | Mild T2 - Mild T3 | -0.16 | -0.63 | 0.30 | 0.98 |
|  | Mild T2 - Moderate T2 | -0.11 | -0.77 | 0.55 | 1.00 |
|  | Mild T2 - Severe T2 | -0.62 | -1.54 | 0.31 | 0.50 |
|  | Mild T3 - Moderate T3 | 0.11 | -0.57 | 0.78 | 1.00 |
|  | Mild T3 - Severe T3 | -0.39 | -1.36 | 0.58 | 0.94 |
|  | Moderate T1 - Moderate T2 | -0.60 | -1.36 | 0.16 | 0.25 |
|  | Moderate T1 - Moderate T3 | -0.55 | -1.34 | 0.25 | 0.43 |
|  | Moderate T1 - Severe T1 | -0.50 | -1.41 | 0.42 | 0.75 |
|  | Moderate T2 - Moderate T3 | 0.05 | -0.67 | 0.78 | 1.00 |
|  | Moderate T2 - Severe T2 | -0.51 | -1.49 | 0.48 | 0.80 |
|  | Moderate T3 - Severe T3 | -0.49 | -1.56 | 0.57 | 0.87 |
|  | Severe T1 - Severe T2 | -0.61 | -1.62 | 0.40 | 0.62 |
|  | Severe T1 - Severe T3 | -0.55 | -1.61 | 0.52 | 0.80 |
|  | Severe T2 - Severe T3 | 0.06 | -1.07 | 1.20 | 1.00 |
| *LV 4ch EF (%)* | Mild T1 - Mild T2 | -0.29 | -2.73 | 2.15 | 1.00 |
|  | Mild T1 - Mild T3 | 0.71 | -1.66 | 3.08 | 0.99 |
|  | Mild T1 - Moderate T1 | -0.42 | -3.87 | 3.02 | 1.00 |
|  | Mild T1 - Severe T1 | -0.92 | -4.57 | 2.74 | 1.00 |
|  | Mild T2 - Mild T3 | 1.00 | -1.36 | 3.37 | 0.92 |
|  | Mild T2 - Moderate T2 | 1.18 | -2.04 | 4.40 | 0.97 |
|  | Mild T2 - Severe T2 | 0.15 | -4.21 | 4.51 | 1.00 |
|  | Mild T3 - Moderate T3 | -1.06 | -4.41 | 2.28 | 0.99 |
|  | Mild T3 - Severe T3 | -2.40 | -7.05 | 2.26 | 0.80 |
|  | Moderate T1 - Moderate T2 | 1.32 | -2.48 | 5.12 | 0.98 |
|  | Moderate T1 - Moderate T3 | 0.07 | -3.87 | 4.02 | 1.00 |
|  | Moderate T1 - Severe T1 | -0.49 | -4.78 | 3.79 | 1.00 |
|  | Moderate T2 - Moderate T3 | -1.25 | -5.00 | 2.51 | 0.98 |
|  | Moderate T2 - Severe T2 | -1.04 | -5.77 | 3.70 | 1.00 |
|  | Moderate T3 - Severe T3 | -1.33 | -6.50 | 3.84 | 1.00 |
|  | Severe T1 - Severe T2 | 0.78 | -4.11 | 5.66 | 1.00 |
|  | Severe T1 - Severe T3 | -0.77 | -5.93 | 4.40 | 1.00 |
|  | Severe T2 - Severe T3 | -1.54 | -7.14 | 4.06 | 0.99 |
| *LV EDVi 4ch (ml/m2)* | Mild T1 - Mild T2 | 0.13 | -6.09 | 6.34 | 1.00 |
|  | Mild T1 - Mild T3 | -2.52 | -8.43 | 3.39 | 0.92 |
|  | Mild T1 - Moderate T1 | -12.00 | -21.84 | -2.16 | 0.01 |
|  | Mild T1 - Severe T1 | -26.97 | -37.74 | -16.20 | <0.001 |
|  | Mild T2 - Mild T3 | -2.65 | -8.39 | 3.09 | 0.88 |
|  | Mild T2 - Moderate T2 | -10.77 | -19.74 | -1.80 | 0.01 |
|  | Mild T2 - Severe T2 | -34.31 | -46.98 | -21.64 | <0.001 |
|  | Mild T3 - Moderate T3 | -13.88 | -23.14 | -4.62 | <0.001 |
|  | Mild T3 - Severe T3 | -48.09 | -61.40 | -34.79 | <0.001 |
|  | Moderate T1 - Moderate T2 | 1.36 | -8.15 | 10.86 | 1.00 |
|  | Moderate T1 - Moderate T3 | -4.40 | -14.35 | 5.54 | 0.90 |
|  | Moderate T1 - Severe T1 | -14.97 | -26.96 | -2.98 | <0.001 |
|  | Moderate T2 - Moderate T3 | -5.76 | -14.87 | 3.35 | 0.56 |
|  | Moderate T2 - Severe T2 | -23.53 | -36.55 | -10.51 | <0.001 |
|  | Moderate T3 - Severe T3 | -34.21 | -48.34 | -20.08 | <0.001 |
|  | Severe T1 - Severe T2 | -7.21 | -19.81 | 5.39 | 0.69 |
|  | Severe T1 - Severe T3 | -23.64 | -36.99 | -10.30 | <0.001 |
|  | Severe T2 - Severe T3 | -16.44 | -30.54 | -2.33 | 0.01 |
| *LV ESVi 4ch (ml/m2)* | Mild T1 - Mild T2 | 0.22 | -2.69 | 3.14 | 1.00 |
|  | Mild T1 - Mild T3 | -1.26 | -4.03 | 1.51 | 0.89 |
|  | Mild T1 - Moderate T1 | -4.61 | -9.02 | -0.20 | 0.03 |
|  | Mild T1 - Severe T1 | -10.53 | -15.31 | -5.75 | <0.001 |
|  | Mild T2 - Mild T3 | -1.49 | -4.18 | 1.21 | 0.73 |
|  | Mild T2 - Moderate T2 | -5.48 | -9.47 | -1.50 | <0.001 |
|  | Mild T2 - Severe T2 | -12.54 | -18.28 | -6.81 | <0.001 |
|  | Mild T3 - Moderate T3 | -5.05 | -9.13 | -0.97 | <0.001 |
|  | Mild T3 - Severe T3 | -18.17 | -24.03 | -12.32 | <0.001 |
|  | Moderate T1 - Moderate T2 | -0.65 | -5.09 | 3.78 | 1.00 |
|  | Moderate T1 - Moderate T3 | -1.70 | -6.32 | 2.91 | 0.97 |
|  | Moderate T1 - Severe T1 | -5.92 | -11.36 | -0.47 | 0.02 |
|  | Moderate T2 - Moderate T3 | -1.05 | -5.29 | 3.19 | 1.00 |
|  | Moderate T2 - Severe T2 | -7.06 | -13.06 | -1.06 | 0.01 |
|  | Moderate T3 - Severe T3 | -13.12 | -19.47 | -6.78 | <0.001 |
|  | Severe T1 - Severe T2 | -1.80 | -7.81 | 4.22 | 0.99 |
|  | Severe T1 - Severe T3 | -8.91 | -15.09 | -2.73 | <0.001 |
|  | Severe T2 - Severe T3 | -7.11 | -13.84 | -0.39 | 0.03 |

*Means adjusted for age, sex, ethnicity and geographic location. Left atrium (LA), four chamber (4ch) posterior long axis view (PLAX), mitral valve (MV), anterior (Ant.), interventricular septum (IVS), left ventricular end diastolic diameter in posterior long axis view (LVEDD), left ventricular posterior wall diameter (LVPWD), left ventricular end systolic volume in posterior long axis view (LVESD), left ventricular (LV), end diastolic volume indexed to BSA (EDVi), end systolic volume indexed to BSA (ESVi), ejection fraction (EF), confidence interval (CI), T1 = timepoint 1, T2 = timepoint 2, T3 = timepoint 3.*

#### *Supplementary Table 3:* *Adjusted means with 95% confidence intervals for LV parameters based on linear mixed effect models demonstrate larger volumes in severe rheumatic heart disease.*

| **Measurement** | **Group and timepoint** | **Mean estimate** | **Lower CI** | **Upper CI** |
| --- | --- | --- | --- | --- |
| *LVEDD Z score* | Mild T1 | 0.13 | -0.28 | 0.53 |
|  | Mild T2 | 0.17 | -0.23 | 0.57 |
|  | Mild T3 | -0.21 | -0.60 | 0.17 |
|  | Moderate T1 | 1.63 | 1.14 | 2.11 |
|  | Moderate T2 | 0.93 | 0.49 | 1.38 |
|  | Moderate T3 | 0.53 | 0.06 | 1.00 |
|  | Severe T1 | 2.55 | 2.02 | 3.07 |
|  | Severe T2 | 2.65 | 2.04 | 3.27 |
|  | Severe T3 | 3.65 | 2.98 | 4.32 |
| *LVESD Z score* | Mild T1 | 0.05 | -0.32 | 0.41 |
|  | Mild T2 | 0.08 | -0.28 | 0.44 |
|  | Mild T3 | -0.10 | -0.44 | 0.24 |
|  | Moderate T1 | 1.09 | 0.65 | 1.53 |
|  | Moderate T2 | 0.63 | 0.23 | 1.02 |
|  | Moderate T3 | 0.16 | -0.27 | 0.58 |
|  | Severe T1 | 1.56 | 1.09 | 2.03 |
|  | Severe T2 | 2.01 | 1.45 | 2.56 |
|  | Severe T3 | 2.29 | 1.69 | 2.90 |
| *LA area 4ch (cm^2^)* | Mild T1 | 12.66 | 11.32 | 14.01 |
|  | Mild T2 | 13.01 | 11.66 | 14.35 |
|  | Mild T3 | 14.83 | 13.53 | 16.14 |
|  | Moderate T1 | 15.83 | 14.26 | 17.41 |
|  | Moderate T2 | 15.76 | 14.26 | 17.26 |
|  | Moderate T3 | 18.34 | 16.76 | 19.92 |
|  | Severe T1 | 17.56 | 15.85 | 19.27 |
|  | Severe T2 | 19.66 | 17.66 | 21.66 |
|  | Severe T3 | 22.33 | 20.18 | 24.49 |
| *MV annulus Z score* | Mild T1 | 2.31 | 2.20 | 2.43 |
|  | Mild T2 | 2.35 | 2.24 | 2.46 |
|  | Mild T3 | 2.57 | 2.46 | 2.68 |
|  | Moderate T1 | 2.46 | 2.32 | 2.60 |
|  | Moderate T2 | 2.46 | 2.33 | 2.59 |
|  | Moderate T3 | 2.68 | 2.54 | 2.82 |
|  | Severe T1 | 2.51 | 2.35 | 2.66 |
|  | Severe T2 | 2.80 | 2.61 | 2.98 |
|  | Severe T3 | 2.89 | 2.69 | 3.09 |
| *IVS Z score* | Mild T1 | -1.14 | -1.42 | -0.86 |
|  | Mild T2 | -0.72 | -0.99 | -0.45 |
|  | Mild T3 | -0.71 | -0.96 | -0.45 |
|  | Moderate T1 | -0.74 | -1.10 | -0.37 |
|  | Moderate T2 | -0.45 | -0.78 | -0.13 |
|  | Moderate T3 | -0.63 | -0.98 | -0.28 |
|  | Severe T1 | -0.26 | -0.66 | 0.14 |
|  | Severe T2 | 0.04 | -0.44 | 0.51 |
|  | Severe T3 | -0.66 | -1.17 | -0.15 |
| *LVPWD Z score* | Mild T1 | -0.94 | -1.27 | -0.61 |
|  | Mild T2 | -0.58 | -0.90 | -0.26 |
|  | Mild T3 | -0.42 | -0.72 | -0.12 |
|  | Moderate T1 | -1.07 | -1.50 | -0.65 |
|  | Moderate T2 | -0.47 | -0.85 | -0.09 |
|  | Moderate T3 | -0.52 | -0.93 | -0.12 |
|  | Severe T1 | -0.58 | -1.04 | -0.11 |
|  | Severe T2 | 0.04 | -0.52 | 0.59 |
|  | Severe T3 | -0.03 | -0.63 | 0.57 |
| *LV 4ch EF (%)* | Mild T1 | 60.93 | 59.46 | 62.41 |
|  | Mild T2 | 61.22 | 59.75 | 62.69 |
|  | Mild T3 | 60.22 | 58.81 | 61.62 |
|  | Moderate T1 | 61.36 | 59.40 | 63.32 |
|  | Moderate T2 | 60.04 | 58.22 | 61.86 |
|  | Moderate T3 | 61.28 | 59.33 | 63.24 |
|  | Severe T1 | 61.85 | 59.70 | 64.00 |
|  | Severe T2 | 61.07 | 58.48 | 63.67 |
|  | Severe T3 | 62.62 | 59.78 | 65.45 |
| *LV EDVi 4ch (ml/m^2^)* | Mild T1 | 45.61 | 40.37 | 50.85 |
|  | Mild T2 | 45.48 | 40.29 | 50.67 |
|  | Mild T3 | 48.13 | 43.18 | 53.08 |
|  | Moderate T1 | 57.61 | 51.33 | 63.89 |
|  | Moderate T2 | 56.25 | 50.48 | 62.02 |
|  | Moderate T3 | 62.01 | 55.92 | 68.10 |
|  | Severe T1 | 72.58 | 65.77 | 79.38 |
|  | Severe T2 | 79.79 | 71.86 | 87.71 |
|  | Severe T3 | 96.22 | 87.72 | 104.72 |
| *LV ESVi 4ch (ml/m^2^)* | Mild T1 | 17.59 | 15.50 | 19.68 |
|  | Mild T2 | 17.37 | 15.30 | 19.43 |
|  | Mild T3 | 18.85 | 16.91 | 20.80 |
|  | Moderate T1 | 22.20 | 19.57 | 24.83 |
|  | Moderate T2 | 22.85 | 20.47 | 25.23 |
|  | Moderate T3 | 23.90 | 21.38 | 26.42 |
|  | Severe T1 | 28.12 | 25.24 | 30.99 |
|  | Severe T2 | 29.91 | 26.44 | 33.38 |
|  | Severe T3 | 37.02 | 33.38 | 40.67 |

Left atrium (LA), four chamber (4ch) posterior long axis view (PLAX), mitral valve (MV), anterior (Ant.), interventricular septum (IVS), left ventricular end diastolic diameter in posterior long axis view (LVEDD), left ventricular posterior wall diameter (LVPWD), left ventricular end systolic volume in posterior long axis view (LVESD), left ventricular (LV), end diastolic volume indexed to BSA (EDVi), end systolic volume indexed to BSA (ESVi), ejection fraction (EF), confidence interval (CI), T1 = timepoint 1, T2 = timepoint 2, T3 = timepoint 3.

*Supplementary Table 4: Inter-observer variability over 20 randomly selected studies.*

| *Parameters* | *Correlation coefficient* | *Intraclass coefficient* |
| --- | --- | --- |
| *LA area 4ch (cm^2^)* | 0.89 | 0.74 |
| *MV annulus (cm)* | 0.86 | 0.59 |
| *LV EDV 4ch (ml/m^2^)* | 0.92 | 0.92 |
| *LV ESV 4ch (ml/m^2^)* | 0.81 | 0.87 |

*LA = left atriaum; 4ch = 4-chamber-view on echocardiogram; MV = mitral valve; LV = left ventricle; EDV = end-diastolic volume; ESV = end-systolic volume*
